# Supplementary material for: Chromothripsis during telomere crisis is independent of NHEJ, and consistent with a replicative origin
Source: Genome Res. 2019 May;29(5):737–49. doi: 10.1101/gr.240705.118 (PMC6499312; doi:10.1101/gr.240705.118)
Supplement: Supplemental Material [file supp_gr.240705.118_Supplemental_file_1.zip › contigs/annotated_contigs/DB51/contig.2.DB51_length_363_mean_cov_3.97796143251.docx]

**DB51_length_363_mean_cov_3.97796143251**

```
C|TTATTTATCCCTTTGCCAATATTATATTGCCTTAATTCATGGAGCTTTTTATTATTATATTCAGTAAGACAAATTCCTATATCTGTT
  >chr6:79336285-79336461 + E=1e-91
TCTTCTTCAGGAAAGTTTTGGTTATTCTTGAGCCTTTATGTTTTCATTTAAATTTTAAAACCAACTTGTCAAGTTCCTTAAAAAAAAAA

|AG|TTCACTTCTAGGAAAGGCATTGGGAACCCTTGATGTTGCCCCTGCCGCGAAGCTCTCTTTCACACTCCCGGCCGAAAGCCCTCAG
   >chr6:14964768-14964952 - E=2e-99
AAGATGCGAGGAGGAGGATTTTGGCATGGACAGTGATGCCATAGGGCAGTTGCTGTGGTAACAGGAGTCTCTCATGCTGTTGGAGTAGT

GTCTGGGAGT
```
